# Radiation Effects of Mobile Phones and Tablets on the Skin: A Systematic Review

**DOI:** 10.1155/2018/9242718

**Published:** 2018-04-12

**Authors:** A. Keykhosravi, M. Neamatshahi, R. Mahmoodi, E. Navipour

**Affiliations:** ^1^Department of Pediatrics, Faculty of Medicine, Sabzevar University of Medical Sciences, Sabzevar, Khorasan Razavi, Iran; ^2^Department of Social Medicine, Faculty of Medicine, Research Center Social Determinants health, Sabzevar University of Medical Sciences, Khorasan Razavi, Iran; ^3^Department of Social Medicine, Faculty of Medicine, Sabzevar University of Medical Sciences, Sabzevar, Khorasan Razavi, Iran

## Abstract

**Background:**

Skin health has become a worldwide concern. Most of the studies investigated the effect of mobile phone radiation on DNA and animals, but a few studies were carried out about skin diseases in mobile phone and tablet users. Few systematic studies have examined the relationship between mobile phone exposure and skin diseases.

**Methods:**

We evaluated the association between mobile phones and tablets and skin diseases. We checked databases including PubMed, Scopus, Springer, Cochrane, and Google Scholar from 1995 to 2013. The eligibility criteria were descriptive, and observational studies were in English and Persian language, and the subjects were of all ages and reported skin disease.

**Results:**

Most of the studies focused on signs and less on skin cancer. In total, 6 studies were included with 392119 participants with age over 25 years. In a nationwide cohort study in Denmark for BCC, the IRR (incidence rate ratios) estimates remained near unity among men and women. In the other studies, they reported an increase in temperature, hypersensitivity of warmth, facial dermatitis, angiosarcoma of the scalp, and burning sensations in the facial skin after mobile phone use on the exposed side and more within the auricle and behind/around the ear.

**Conclusions:**

Overall evaluations showed that the level of evidence associated with the effects of radiation from the mobile phone and tablet on the skin is poor. This review shows a necessity for more studies in this area.

## 1. Introduction

Mobile phones and tablets have become the most effective communication tools especially in metropolitan cities [1]. Exposure of the general population to radiofrequency (RF) fields from mobile phones and other communication tools has become universal and continuous in recent years [2]. The number of mobile phone users has gone up to 5 billion in a world of 7.4 billion [1]. Development of using mobile phones has increased concerns about the safety of health, in recent years. The studies reflected public concerns about childhood and adult cancers. The possibility that some individuals experience hypersensitivity or other symptoms in response to mobile exposure was a high priority for research [3].

The emitted radiation in mobile phone and tablet is electromagnetic ray in the microwave range (850–1800) [1]. Collected evidence indicates that the frequency produced by mobile phones or base stations may affect the health of the people [4, 5].

The skin receives much radiation in contact with mobile phone and tablet although many studies have been carried out on the effect of electromagnetic radiation on biologic system and intracranial tumors [1, 6], Diseases of the skin, especially skin cancers and contact dermatitis, are very important because of their high prevalence, chronic nature of the disease, and high impact on the quality of life [7] (skin diseases cause pain and discomfort in 21% to 87% of the affected people) [8]. Skin diseases allocated high burden of disease (rank eighteenth) in all age groups [9].

Among the factors that are related to skin diseases, less attention has been paid to environmental factors. Most studies have been done on these factors, in animals. The results indicate that exposure to radiation emitted by mobile phones caused skin changes in rats, as, increased thickness of surface layer, atrophy of epidermis, deep layer proliferation, vascular proliferation, impairment in collagen tissue and protein expression in human skin in proteomics approach.

The lack of studies on the association between mobile phone use and risk of skin diseases prompted us to examine these associations in this systematic review.

## 2. Methods

### 2.1. Search Strategy

We reviewed PubMed, Scopus, Cochrane library, Google Scholar, and Springer 1995 to 2017. A range of mesh, key words, and their combinations were used, including skin disease, cell phone, smart phone, mobile phone, electrochemical magnetic field, skin cancer, skin carcinoma, and health effects. We also did not consider articles that merely assessed the physiologic effects and reviewed the bibliographies for additional publications. The language of publication was English and Persian.

### 2.2. Selection Criteria

We included studies that had the following criteria: cross-sectional, cohort, and crossover studies to refer to the impact of mobile phone radiation on skin diseases. If the data were duplicated, the first published study was entered in the analysis. Studies retrieved from the databases that had the predetermined selection criteria were assessed by two of the authors independently. If the authors had disagreed, they resolved by discussion or in consultation with a third author.

The exclusion criteria were included: nonrelevant articles on the type of study and subject of research, low-quality studies based on the CASP checklist, and studies that did not contain enough information. In the larger studies included in this survey were any conducted solely by telephone.

### 2.3. Quality Evaluation of Articles

The quality of the articles was evaluated based on the CASP scale by two researchers. This checklist contains 11 sections for cross-sectional studies and 12 sections for cohort studies.

### 2.4. Data Extraction

The information consisted of the name of the first author, where (country) the study was conducted, and the date of publication, sample size, and the method of data collection (Table 1).

## 3. Results

**Figure 1:**
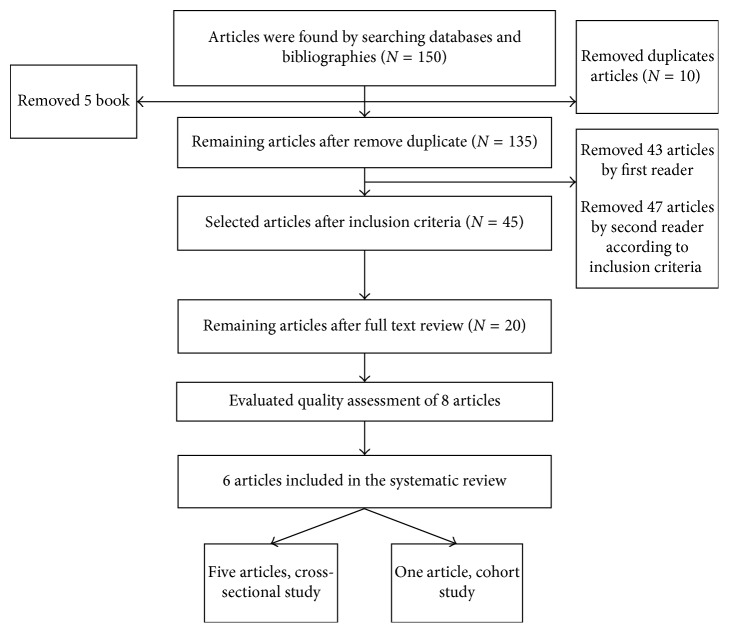
Flow chart of the process of study entering in a systematic review study.

Figure 1 shows a flow chart that we searched and selected appropriate articles. In the first step, a total of 150 articles were found by searching databases and bibliographies. 75 articles were excluded because those had not inclusion criteria. We reviewed the full text of selected articles, as shown in Figure 1, 6 articles were included in the systematic review.

The sample size in the 6 studies (one cohort study and five cross-sectional studies) was 392119, and the details of the articles are presented in Table 1. All studies were done on both sexes and the mean age was 35 years. The diseases that were assessed in these studies included skin cancer, dermatitis, itching, warmth and burning feeling, and rash (Table 2).

1. In a nationwide cohort study, 355,701 mobile phone users in Denmark from 1987 to 2007 were followed up. After a period of at least 20 years, little evidence of skin cancer risk was observed among the mobile phone users [6].
2. In a cross-sectional study that was conducted in 2008 on 2000 Swedish adolescence aged 15–19 years, the most participants assessed, they had skin complaints as rash and dermatitis [10].
3. A cross-sectional study was initiated in 1995 including 11982 GSM and NMT users in Sweden and 2500 in Norway, the authors observed a low-risk warmth on the behind and around the ear [11].
4. A cross-sectional study on 17,000 people in Norway and Sweden showed 31% mobile phone users in Norway, and 13% of those in Sweden had experienced at least one symptom that included the sensations of warmth on the ear and behind/around the ear, burning sensations in the facial skin. Most skin symptoms usually began during or after the call and lasted for up to 2 h but these results suggest an awareness of the symptoms, but not necessarily a serious health problem [12].
5. A survey was conducted among a total of 330 medical students at the King Saud University. This study presents an overview about the impact of radiofrequency waves on the health of medical students in Saudi Arabia. The most of skin symptoms were reported including facial dermatitis [13].

## 4. Conclusions

In this systematic review, we searched articles in databases. Abstracts and text of the articles were examined from various aspects. Eight articles were evaluated for quality, and then, six papers entered a systematic review.

In the present study, the studied population was of both sexes with an average age of 35 years; therefore, this shows the importance of the issue. These persons have high performance in communities, so their illness increases the burden of the disease.

All studies were cross-sectional or cohort, and there were different morbidity indexes between mobile radiation and skin diseases. The duration of exposure to mobile radiation was very different for skin signs; therefore, we did not have the possibility of meta-analyzing studies. Oftedal et al. in 1999, with a sample size of 17,000, reported the prevalence of skin problems caused by mobile use [12] However, in a cohort study, Poulsen et al. reported the incidence of skin cancer [6].

We found that the use of mobile phones was associated with a mildly increased risk of skin problems. This is the first systematic review on the effects of mobile phone radiation on skin diseases.

From 6 articles that enter to systematic review, two studies did not reveal a serious health problem [12, 13]. In two other studies, these pointed to warmth sensation after the use of mobile phones [6, 11]. Cell phones play an important role in people's lives since the last few decades, and people have been exposed for so long, so addressing their effects on health can prevent harmful effects among mobile phone users. IARC (International Agency for Research on Cancer) classified that the mobile-emitted radiation could be some risk of carcinogenicity, so further studies into heavy use of mobile phones needs to be conducted [14].

Previous studies have reported that collagen tissue increased in cells exposed to mobile radiation. Mobile phone radiation for one hour causes morphological changes and increased fibroblast activity of the skin. Another study also found that exposure to 900 MHz mobile phone radiation creates exocytosis in skin cells. Some studies have shown that the degree of destruction caused by mobile radiation is related to the duration of exposure to radiation [4]. Epidemiological studies conducted on humans and animals indicate that electromagnetic waves produce a wide range of side effects in different systems of the body [15], but they have not achieved a definite result [16]. Skin reactions in the ears and around it are the most common symptoms reported among mobile phone users during a telephone call [12, 17]. As worldwide rates of mobile phone users rise, Richardson showed that mobile phone-associated contact dermatitis is increasing. In order to control allergens in phones, many phones have metals such as nickel that are sufficient to induce contact dermatitis; Therefore, patients with dermatitis of the face, neck, hands, breasts, or anterior thighs, should be examined for exposure to mobile [18].

In patients with profuse sweating, it provides a predisposing condition, and the penetration of nickel to the skin increases the occurrence of contact dermatitis. Therefore, mobile phone dermatitis should be considered in the differential diagnosis of contact dermatitis. The patch test and dimethylglyoxime test may be helpful in establishing the diagnosis [19].

In the study of Hardel et al. in 2011, he concluded that there is no relationship between the use of mobile phones and skin cancer [20].

Overall evaluations showed that the effects of mobile phone radiation on skin diseases are weak and have no statistical significance. Some studies have shown weak impacts, and some studies have found that over ten years of mobile use have been effective, but mobile phones are still a new technology and little evidence about long-term side effects is available, as a result, prevention is the best approach. Epidemiological studies on this topic are limited, and its long-term effects have not been evaluated, and there is a gap in the assessment relationship between mobile phone radiation and skin diseases. People are worried about the health effects of mobile phones, especially since it is part of daily life. As a result, the implementation of standard policies and strategic planning for primary health care by government officials on this topic is necessary to reduce people's concerns in order to provide suitable solutions for high-risk people. These programs require extended studies on mobile phone technology and its impact on the safety of mobile users. Our study has limitations. First, only few studies on the study of mobile phone radiation and skin disease are available. Second, we did not have the ability to access the full text of some articles and low levels of evidence.

## Figures and Tables

**Table 1:** Studies on the effects of mobile on skin diseases.

First author	Date of publication	Date of performance	Country	Age (range)	Participants	Place and population
Sandström et al. [11]	2000	1995	Sweden Norway	30–50	17000	Norway and Sweden
Oftedal et al. [12]	2000	1999	Sweden	30–50	17000	Norway and Sweden
Fredrik Soderqvist	2008	2005–2006	Sweden	15–19	2000	Among Swedish adolescents
Khan [13]	2008	2007	Saudi Arabia	NA	330	Medical students in Saudi Arabia
Poulsen et al. [6]	2012	1990–2007	Denmark	30	355701	CANULI (“cancer of social ulighed”; cancer and social inequality)
Claudio Gómez-Perretta	2013	2003	Spain	15–81	88	Known illness in 2003 was subsequently disregarded

**Table 2:** Methodology of studies included in the systematic review.

First author	Study design	Statistical methods	Outcome	Exposure	Measure of association	Conclusions
Sandström et al. [11]	Cross-sectional	Multivariate logistic regression	Warming and burning ear skin	Cellular phone	Prevalence of symptoms	A statistically significant association between calling time/number of calls per day and the prevalence of warmth behind/around or on the ear, headaches, and fatigue
Oftedal et al. [12]	Cross-sectional	Chi-square test	Burning sensations in the facial skin and warming sensation behind and around the ear	Mobile phone	Percentage of symptoms (%)	These findings, together with our results, may indicate a causal relation between the use of mobile phones and warming sensations. These results suggest an awareness of the symptoms, but no serious skin health problem
Fredrik Soderqvist	Cross-sectional	Chi-square test and logistic regression	Health symptoms	Wireless telephones	Odds ratio	The findings of the present study indicate that the use of mobile phones causes skin rash & burning sensation
Khan [13]	Cross-sectional	Chi-square test	Facial dermatitis	Mobile phone	Percentage of health complaints	The findings of the present study indicate that mobile phones play a large part in the daily life of medical students, and therefore its impact on psychology and health should be discussed with the students to prevent the harmful effects
Poulsen et al. [6]	Cohort	Log-linear Poisson regression models	Skin cancer	Mobile phone	Incidence rate ratios (IRRs)	This nationwide study of mobile phone subscribers provided no support for a relationship between mobile phone use and skin cancer.
Claudio Gómez-Perretta	Cross-sectional	Analysis of variance test (ANOVA)	Health symptoms	Mobile phone	Odds ratio	The findings of this study indicate that no relationship was found between the rays emitted from mobile phone and the skin changes
